# Association Between Side of Living Kidney Donation and Post-Transplant Outcomes

**DOI:** 10.3389/ti.2022.10117

**Published:** 2022-04-04

**Authors:** Ellen L. K. Dobrijevic, Eric H. K. Au, Natasha M. Rogers, Philip A. Clayton, Germaine Wong, Richard D. M. Allen

**Affiliations:** ^1^ Faculty of Medicine and Health, University of Sydney, Sydney, NSW, Australia; ^2^ The Centre for Transplant and Renal Research, Westmead Institute of Medical Research, Westmead, NSW, Australia; ^3^ Department of Renal and Transplant Medicine, Westmead Hospital, Westmead, NSW, Australia; ^4^ Starzl Transplant Institute, University of Pittsburgh, Pittsburgh, PA, United States; ^5^ Central and Northern Adelaide Renal and Transplantation Services, Adelaide, SA, Australia; ^6^ Australia and New Zealand Dialysis and Transplant (ANZDATA) Registry, Adelaide, SA, Australia; ^7^ Discipline of Medicine, University of Adelaide, Adelaide, SA, Australia; ^8^ Department of Transplantation Surgery, Westmead Hospital, Westmead, NSW, Australia

**Keywords:** patient survival, kidney transplant, living donor, graft survival, delayed graft function

## Abstract

**Background:** Right-sided living donor kidneys have longer renal arteries and shorter veins that make vascular anastomosis more challenging. We sought to determine whether recipients of right-sided living donor kidneys have worse outcomes than left-sided kidney recipients.

**Methods:** An observational analysis of the Australia and New Zealand Dialysis and Transplant Registry (ANZDATA) was undertaken. We used adjusted logistic regression to determine the association between side and delayed graft function (DGF) and time-stratified adjusted cox regression models for graft and patient survivals.

**Results:** Between 2004 and 2018, 4,050 living donor kidney transplants were conducted with 696 (17.2%) using right kidneys. With reference to left kidneys, the adjusted OR (95% CI) for DGF was 2.01 (1.31–3.09) for recipients with right kidneys. Within 30 days, 46 allografts (1.4%) were lost, with major causes of overall graft loss being technical, primary non-function and death. Recipients of right donor kidneys experienced a greater risk of early graft loss (aHR 2.02 [95% CI 1.06–3.86], *p* = 0.03), but not beyond 30 days (aHR 0.97 [95% CI 0.80–1.19], *p* = 0.8]).

**Conclusion:** Technical challenge is the most common cause of early graft loss. The risk of early graft loss among recipients who received right kidneys is doubled compared to those who received left living donor kidneys.

## Graphical Abstract

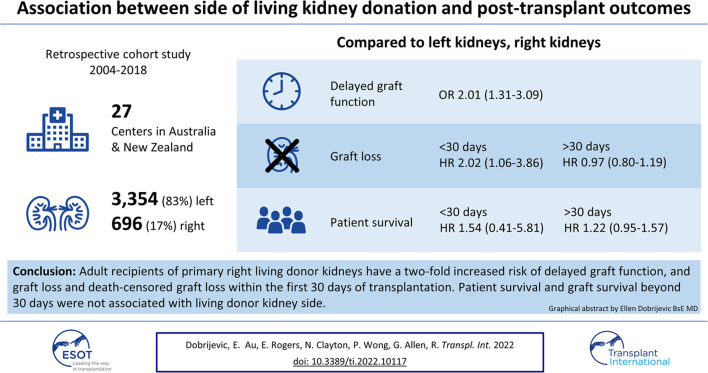

## Introduction

The effect of the side of the living donor kidney on graft and recipient outcomes remains a subject of debate. Transplant surgeons prefer left-sided living donor kidneys because the longer renal vein facilitates implantation of the donor kidney to the deeply situated recipient right iliac vein (1–8). Compared with the use of right living donor kidneys, both the tension on the venous anastomosis and the potential kinking of a longer right renal artery are minimized when using left kidneys (4–6, 9). International registry and cohort studies demonstrate that more left kidneys are transplanted than right, particularly following the introduction of laparoscopic nephrectomy (1, 7, 10, 11). A multicentre analysis of the Organ Procurement and Transplantation Network (OPTN) database between 2000 and 2009 showed approximately 14% of living donor kidneys transplants were right-sided and, with a downward trend over time (2).

The increased technical difficulty of implanting a right donor kidney may predict the greater risk of thrombosis, delayed graft function (DGF) and graft loss for recipients of right compared to left kidneys (2, 9, 12–14). This trend is also observed for deceased donor kidneys (9, 15, 16). A recent systematic review and meta-analysis of observational studies comparing left and right living donor laparoscopic nephrectomies reported that left living donor kidneys had approximately 30% lower rates of DGF and thrombosis compared to right living donor kidneys (13). However, the certainty of the evidence is low, most studies were of small, single centres with substantial heterogeneity in study design, and almost all were judged to have high risk of bias in domains of selection, confounding, and outcomes reporting (13). Furthermore, sensitivity analysis did not demonstrate any significant difference in outcomes between left and right living donor recipients (13). Therefore, this study aimed to assess the association between the side of living donor kidney and patient outcomes including delayed graft function (DGF), early allograft loss and patient survival using data from a large national cohort of kidney transplant recipients.

## Methods

Ethics approval was granted by the Western Sydney Local Health District Human Research Ethics Committee ((6063) 2019/ETH09846) and the ANZDATA executive. This manuscript was prepared following the Strengthening the Reporting of Observational studies in Epidemiology (STROBE) guidelines (17).

### Population

**FIGURE 1:**
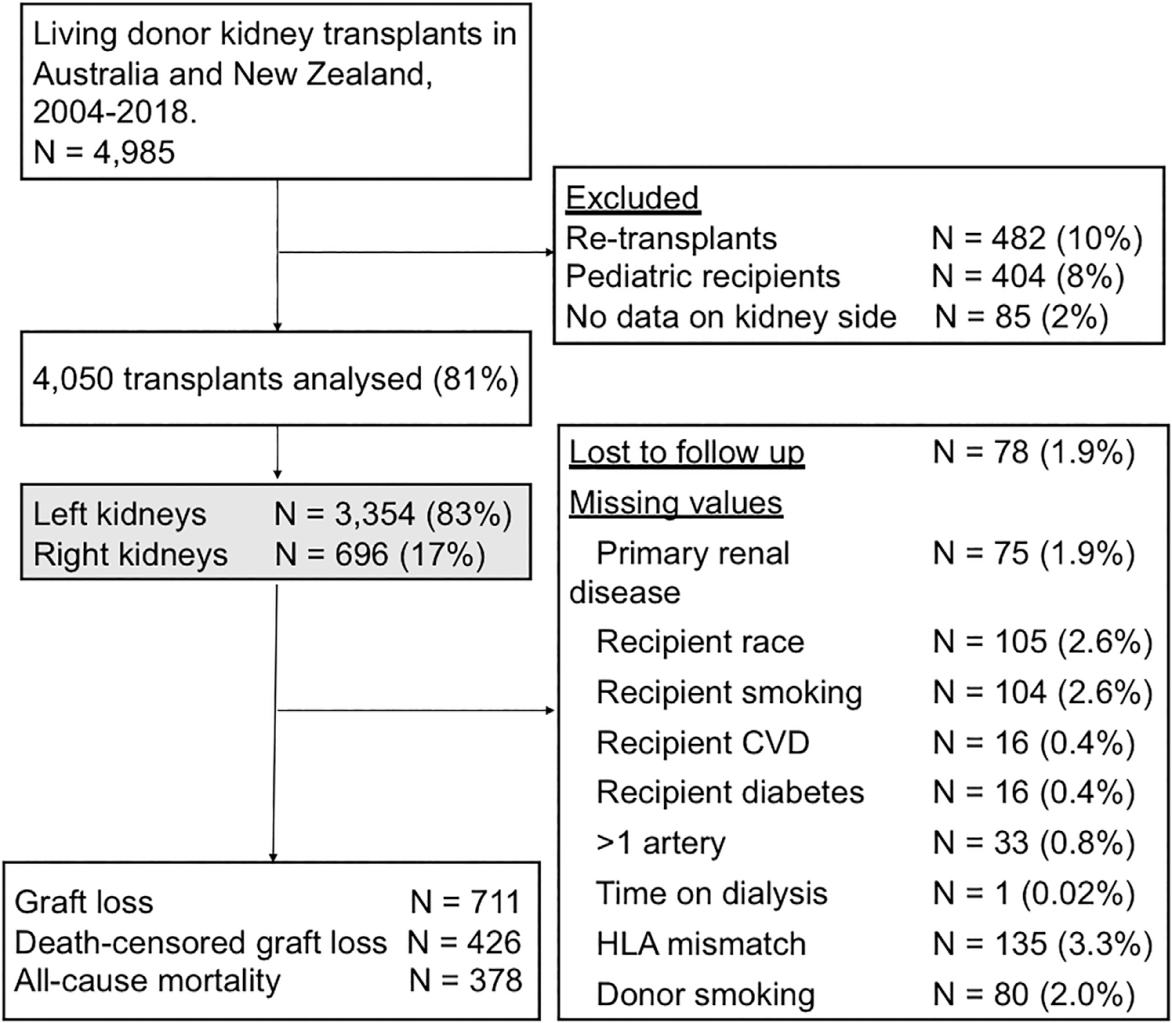
Flow diagram outlining the study cohort and exclusions. CVD, cardiovascular disease; HLA, human leukocyte antigen.

An observational analysis of the Australia and New Zealand Dialysis and Transplant (ANZDATA) registry was undertaken from January 2004 to the end of 2018. Paediatric recipients (404, 8%) and non-primary grafts (482, 10%) were excluded from the analysis, as these populations are expected to have different characteristics and outcome profiles that may be inadequately captured by measured characteristics. Cases with missing data on the key exposure, kidney side, were not included (85, 2%) (Figure 1).

### Data Collection

The key exposure of interest was the side, left or right, of the living donor kidney. Donor baseline characteristics included for analysis were age, sex, ethnicity, smoking, family history of diabetes mellitus, diabetes mellitus, hypertension, and body mass index (BMI).

Recipient baseline characteristics included age, sex, ethnicity, primary kidney disease, smoking, diabetes mellitus, body mass index, cytomegalovirus (CMV) and Epstein-Barr virus (EBV) status, history of chronic lung disease, cardiovascular disease, hepatitis or cancer, and time on dialysis before transplantation. The primary kidney disease of the recipient was classified into glomerulonephritis, polycystic kidney disease, reflux nephropathy, vascular, diabetes mellitus and other. Cardiovascular disease was defined as a history of any one of coronary artery disease, peripheral vascular disease or cerebrovascular disease. Kidney donor surgery and implantation characteristics included the procedure date, donor and recipient relationship, human leukocyte (HLA) mismatches, total ischemia time, number of renal arteries and veins anastomosed, number of ureters, operation type and approach. The relationship between the donor and recipient was classified as related or unrelated. Total ischaemia time was the sum of warm and cold ischemia times, from donor renal artery interruption to the release of the renal artery in the recipient. The information collected by ANZDATA on DGF changed in 2017, from recording grafts requiring dialysis within 72 h to grafts requiring dialysis within 7 days after transplantation. Our analysis therefore defined DGF as recipients who required dialysis within 7 days of transplantation.

### Outcomes

The patient relevant outcomes included in these analyses were overall graft loss, death censored graft loss, all-cause death and DGF. We also sought to compare the cause of early graft loss in the first 30 days after transplantation, between left and right living donor kidneys. Overall graft loss was defined as transplant nephrectomy, recommencing long term dialysis, re-transplantation or death from any cause. Time to graft loss was the period from the date of transplantation until the date of graft failure or death, with cases censored for loss to follow-up and the end of the study period. For death-censored graft loss, recipients were censored at the time of death, loss to follow-up or the end of the study period, whichever one came first. Patient survival was defined as the time from transplantation until patient death, censored for loss to follow up and the end of the study period.

### Statistical Analysis

Categorical variables are presented as counts with percentages and compared using Pearson chi-square tests. Non-normally distributed continuous variables are presented as medians with interquartile ranges and compared using Mann-Whitney U tests. A Pearson’s product-moment correlation was performed to determine the relationship between the total number of transplants performed at each centre and the percentage of right kidneys transplanted. The 17 centres that performed transplants in 2018 were included to capture the centres that are well established and currently active. *p*-values less than 0.05 were considered statistically significant.

### Association Between Side of the Kidney and Delayed Graft Function

Adjusted binomial logistic modelling was used to determine the association between donor kidney side and DGF. Variables with a *p*-value <0.25 on univariate analysis were included in the initial model, as well as kidney side. We then used a step-wise backward elimination process until the variables with *p* < 0.05 remained in the final model. To examine the effect of the change in definition of DGF in 2017, a two-level categorical variable representing the different periods was constructed from the year of transplant (2004–2016, 2017–2018) and added to the final multivariable model. The binomial logistic regression analysis was then fitted using a random effect model to account for clustering of DGF within centres.

### Overall Graft Survival, Death-Censored Graft Survival and All-Cause Death

Time to event outcomes (overall graft survival, death-censored graft survival and all-cause death) were analysed using the Kaplan-Meier method and differences in survival curves were compared using the log-rank test.

### Association Between Sides of the Kidneys, Overall Graft Loss, Death-Censored Graft Loss and All-Cause Death

Adjusted cox regression modelling was used to assess the association between the side of the kidney and allograft outcomes. For each outcome, the initial multivariable model included variables with a *p*-value of less than 0.25 on univariable analysis. The least significant variables were then removed from the base model using a step-wise backward elimination process until only variables with *p* < 0.05 remained in the final parsimonious model. The linearity of continuous variables was assessed by dividing into categories and examining the trend.

The proportional hazards assumptions of the Cox models considering the whole study period (from 2004 to 2018) were tested and the Schoenfeld residuals were plotted for each variable. There was no deviation from the assumption with the key exposure (side of kidney) for overall graft loss, death-censored graft loss and overall mortality. The models were then fitted with the predetermined division at 30 days. Thirty days was selected to elucidate the differences between early and late recipient outcomes and as a clinically relevant timepoint used in studies investigating early kidney graft loss (2, 18, 19). For each outcome two Cox regression models were fitted. The first model analysed events in the first 30 days after transplantation, censoring events from day 31 onwards. The second model analysed events occurring between day 31 to the end of the study period. Compared to the models analysing the whole time period, the models with the division at 30 days had a better fit by comparing the negative 2 log likelihood values.

To assess the robustness of our results, the Cox proportional hazard regression analyses were also fitted using a random effect model (frailty model) to account for clustering of graft loss and mortality risk within centres. Additionally, a three-level categorical variable for transplant year was constructed (2004–2008, 2009–2013, 2014–2018) and added to the final Cox models to assess for era effects, this term was removed from the model if it was not significant.

Analyses were performed using IBM SPSS v25 (IBM Corp., Armonk, NY, United States) and RStudio (RStudio, PBC. Boston, MA, United States).

## Results

There were 4,985 living donor transplants between 2004 and 2018. After excluding paediatric recipients, non-primary grafts, and donor kidneys with missing data on kidney side, the recipient cohort of 4,050 living donor transplants included 3,354 (82.8%) left kidneys and 696 (17.2%) right kidneys (Figure 1). The baseline characteristics of the donors, recipients and the transplant procedures are shown in Tables 1, 2.

**TABLE 1:** Donor baseline characteristics.

Factor	Left (*n* = 3,354)	Right (*n* = 696)	*p*-value
Age, median (IQR)	51 (43–58)	51 (43–59)	0.50
Sex, male (n, %)	1,396 (41.6)	282 (40.5)	0.59
Ethnicity (n, %)			
Caucasian	2,887 (86.7)	615 (89.3)	0.44
Aboriginal or Torres Strait Islander Peoples	72 (2.2)	10 (0.3)	
Maori	71 (2.1)	10 (0.3)	
Pacific Islander	41 (1.2)	8 (0.2)	
Asian	202 (6.0)	39 (1.2)	
Other	55 (1.7)	7 (0.2)	
Smoking (n, %)			
Never	1976 (60.0)	423 (62.3)	0.36
Current	196 (6.0)	44 (6.5)	
Former	1,119 (34.0)	212 (31.2)	
Diabetes mellitus (n, %)			
Nil	3,284 (99.3)	677 (99.0)	0.44
Type 1	2	0	
Type 2—requiring insulin	1	1	
Type 2—non-insulin requiring	10	1	
Gestational	10	4	
Family history of diabetes mellitus (n, %)	592 (19.3)	128 (20.4)	0.53
Hypertension (n, %)	362 (10.9)	71 (10.3)	0.68
BMI (kg/m^2^), median (IQR)	26.5 (23.9–29.2)	26.2 (23.9–29.0)	0.75

**TABLE 2:** Recipient and transplant characteristics.

Factor	Left (*n* = 3,354)	Right (*n* = 696)	*p*-value
Age, median (IQR)	47.0 (35.0–57.0)	50.0 (36.0–60.0)	0.0011
Sex, male (n, %)	2,155 (64.3)	419 (60.2)	0.045
Primary kidney disease (n, %)			
Glomerulonephritis	1,512 (46.0)	306 (44.6)	0.025
Polycystic	585 (17.8)	115 (16.8)	
Reflux	313 (9.5)	57 (8.3)	
Vascular	157 (4.8)	51 (7.4)	
Diabetes mellitus	274 (8.3)	72 (10.5)	
Other	448 (13.6)	85 (12.4)	
Ethnicity (n, %)			
Caucasian	2,683 (82.2)	576 (84.2)	0.30
Aboriginal or Torres Strait Islander Peoples	27 (0.8)	8 (1.2)	
Maori	92 (2.8)	20 (2.9)	
Pacific Islander	89 (2.7)	22 (3.2)	
Asian	310 (9.5)	47 (6.9)	
Other	61 (1.9)	11 (1.6)	
Smoking (n, %)			
Never	2049 (62.8)	419 (61.5)	0.14
Current	211 (6.5)	33 (4.9)	
Former	1,005 (30.8)	229 (33.6)	
Diabetes mellitus (n, %)			
Nil	2,775 (83.1)	546 (78.6)	0.0019
Type 1	78 (2.3)	20 (2.9)	
Type 2—requiring insulin	234 (7.0)	47 (6.8)	
Type 2—non-insulin requiring	251 (7.5)	82 (11.8)	
Cardiovascular disease (n, %)	516 (15.5)	102 (14.7)	0.60
Chronic lung disease (n, %)	154 (4.6)	41 (5.9)	0.15
Hepatitis (n, %)	30 (1.0)	6 (0.9)	0.89
Cancer ever (n, %)	175 (5.2)	42 (6.0)	0.39
BMI, median (IQR)	25.9 (22.8–29.4)	26.2 (22.8–29.6)	0.40
Time on RRT (years), median (IQR)	6.93 (0–20.7)	6.26 (0–21.6)	0.89
Donor-recipient relationship			
Related	1726 (51.5%)	359 (51.6%)	0.96
Unrelated	1,628 (48.5%)	337 (48.4%)	
Ischemia time (hours), median (IQR)	3.00 (2.00–4.00)	3.00 (2.00–4.00)	0.11
Number of arteries			
1	2,765 (82.9%)	560 (82.0%)	0.050
2	532 (16.0%)	107 (15.7%)	
3	35 (1.1%)	14 (2.1%)	
4	2 (0.1%)	2 (0.3%)	
Number of veins			
1	3,233 (97.1%)	619 (90.6%)	<0.001
2	95 (2.9%)	59 (8.6%)	
3	2 (0.1%)	4 (0.6%)	
4	0	1 (0.1%)	
Number of ureters			
1	3,291 (98.9%)	678 (99.3%)	0.41
2	36 (1.1%)	5 (0.7%)	
Operation type			
Hand assisted laparoscopic	1,145 (34.2%)	278 (40.4%)	<0.001
Laparoscopic	1938 (57.9%)	299 (43.5%)	
Open	267 (8.0%)	111 (16.1%)	
Operation approach			
Extraperitoneal	633 (19.5%)	131 (19.5%)	0.10
Transperitoneal	2,618 (80.5%)	542 (80.5%)	
HLA-A mismatch			
0	747 (23.1%)	161 (23.6%)	0.88
1	1729 (53.4%)	366 (53.7%)	
2	764 (23.6%)	155 (22.7%)	
HLA-B mismatch			
0	463 (14.3%)	113 (16.6%)	0.31
1	1,618 (50.0%)	329 (48.2%)	
2	1,158 (35.8%)	240 (35.2%)	
HLA-DR mismatch			
0	672 (20.8%)	154 (22.6%)	0.48
1	1733 (53.5%)	349 (51.3%)	
2	833 (25.7%)	177 (26.0%)	

There were no differences between left and right kidney donors (Table 1). Recipients of right living donor kidneys were more likely to be older (median age 47 for left compared to 50 years for right kidneys), female and have diabetes mellitus (Table 2). Compared to left donor nephrectomies, right donor nephrectomies were more commonly hand-assisted laparoscopic procedures (34.2% left kidneys compared to 40.4% right kidneys) and open procedures (8.0% left kidneys compared to 16.1% right kidneys) (Table 2).

**FIGURE 2:**
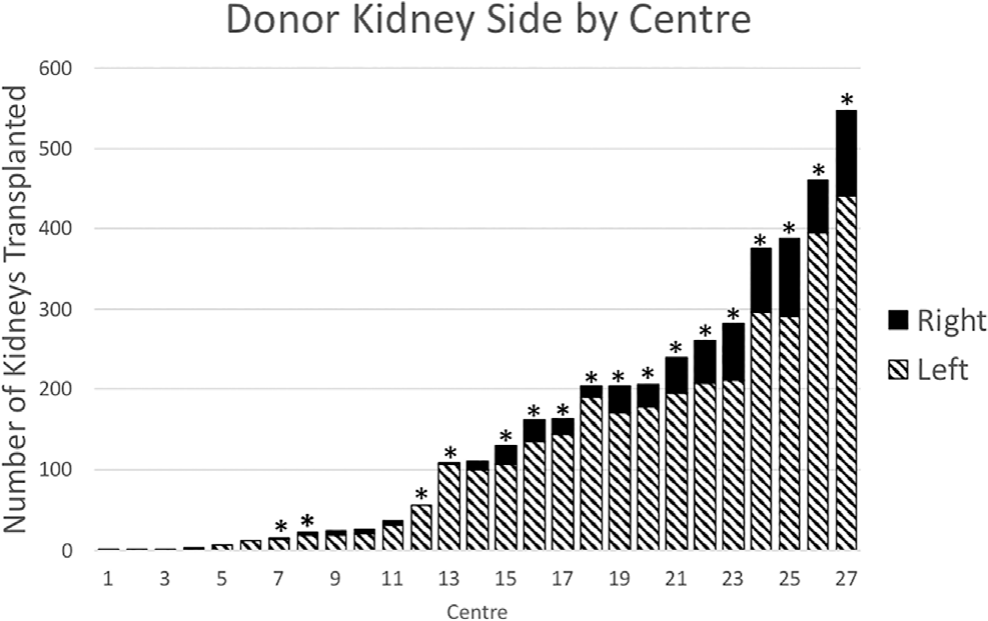
Donor kidney side by transplant centre, 2004–2018. Bars with asterisks (n = 17) indicate centres that performed transplants in 2018.

There was variation in the proportion of right kidneys transplanted between the 27 transplant centres in this study (Pearson chi-square *p* < 0.01) (Figure 2). There was also a positive correlation between the total number of transplants performed at each centre and the percentage of right kidneys transplanted (Pearson’s product-moment correlation r = 0.55, *p* = 0.02). During the time period of the study, 10 of the 27 transplant centres closed or merged with others. Between 2004 and 2018, the proportion the transplanted kidneys each year that were right sided was stable (mean = 17.1%, standard deviation = 2.2%).

### Association Between Kidney Side and Delayed Graft Function

**FIGURE 3:**
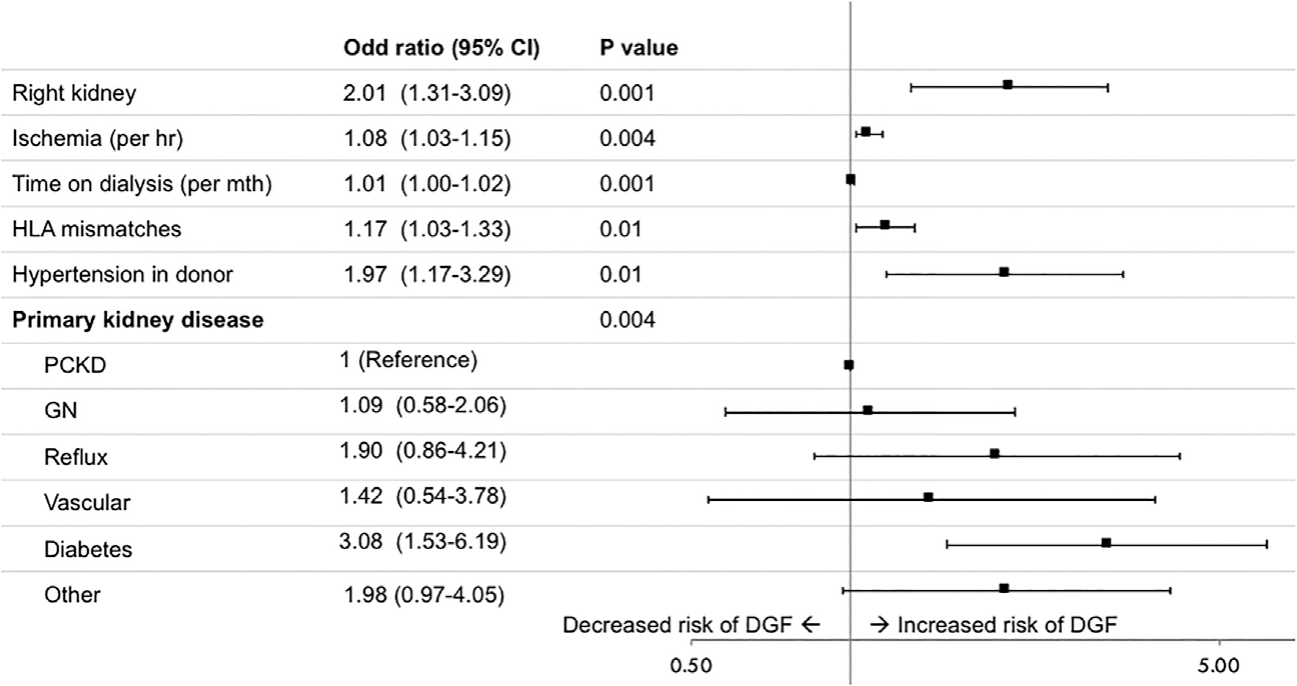
Risk factors for delayed graft function (DGF), defined as the need for dialysis within 7 days of transplantation. GN, glomerulonephritis; PCKD, polycystic kidney disease.

DGF was reported in 3.0% of transplants. Recipients of right kidneys were more likely to experience DGF with 86 recipients (2.6%) of left kidneys compared to 34 (4.9%) of right kidneys affected (*p* = 0.001). Right kidneys were associated with an increased risk of DGF (adjusted odds ratio (OR) (95% CI) 2.01 [1.31–3.09]), adjusting for total ischemia time, time on dialysis before transplant, number of HLA mismatches, donor hypertension and primary kidney disease (Figure 3). In the frailty model, clustering for transplant centre, the adjusted OR (95% CI) for receiving a right compared to left kidney was 2.09 [1.34–3.24]. The risk factor profile for DGF was not significantly altered after accounting for the change in definition of DGF in 2017, with the adjusted OR (95% CI) for receiving a right compared to left kidney being 1.89 (1.24–2.87).

### Causes of All-Cause Graft Loss and Death

Between 2004 and 2018, 736 recipients lost their allografts, with the cause documented in 721 (98.0%) cases (Table 3). During the first 30 days after transplantation, 46 grafts were lost. Right kidneys accounted for 14 (30.4%) of the grafts lost in the first 30 days, despite representing only 17.2% of all transplants. Technical causes (including haemorrhage, renal artery or vein thrombosis and renal artery stenosis) accounted for 50.0% of left kidneys and 28.6% of right kidneys lost. Primary non-function accounted for 9.4% of left kidneys and 35.7% of right kidneys lost in the first 30 days after transplantation (Table 3). After the first 30 days, the main causes of graft loss were death with functioning graft (39.0% left and 42.5% right), followed by chronic allograft nephropathy (31.2% left and 29.1% right) and recurrent glomerulonephritis (10.4% left and 6.0% right) (Table 3). A total of 391 patients died in the study period. The main causes of death were cardiovascular disease (25%), malignancy (23%) and infection (16%). Eleven patients died within the first 30 days of transplantation. Eight were recipients of left LDKs and three were recipients of right kidneys. The main causes of death were cardiac (3 three cases of cardiac arrest of uncertain cause and one case of myocardial infarct) and septicaemia (2 cases).

**TABLE 3:** Causes of graft loss over the study period (from 2004 to 2018) and over the first 30 days after transplant.

Time period	First 30 days after transplantation				Study period (2004–18)			
Side of kidney	Left		Right		Left		Right	
Total	32		14		587		134	
Death with function	6	18.8%	2	14.3%	229	39.0%	57	42.5%
Chronic allograft nephropathy	—	—	—	—	183	31.2%	39	29.1%
Recurrent glomerulonephritis	1	3.1%	0	0%	61	10.4%	8	6.0%
Acute rejection	6	18.8%	3	21.4%	30	5.1%	11	8.2%
Technical	16	50.0%	4	28.6%	25	4.3%	4	3.0%
Primary non-function	3	9.4%	5	35.7%	3	0.5%	5	3.7%
Other	—	—	—	—	56	9.5%	10	7.5%
Pearson chi-square *p*-value				0.2				0.02

### Kaplan-Meier Estimates of Graft, Death-Censored Graft and Patient Survivals

**FIGURE 4:**
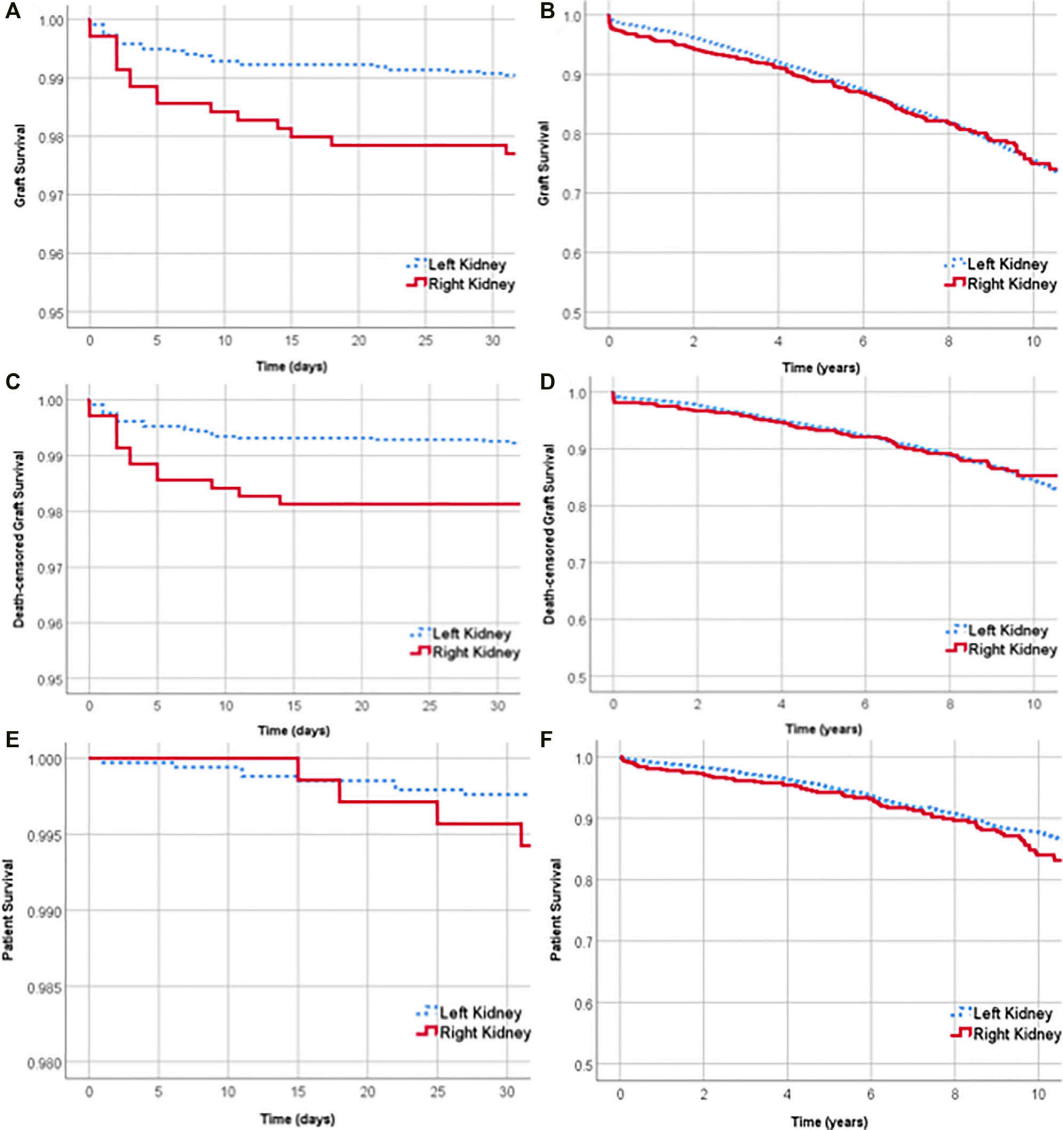
Graft survival of left and right kidneys over the first 30 days after transplant **(A)** and over 10 years **(B)**. Death-censored graft survival over the first 30 days after transplant **(C)** and over 10 years **(D)**. Survival of recipients of left and right living donor kidneys over the first 30 days after transplant **(E)** and over 10 years **(F)**.

Graft survival was lower for right living donor kidney recipients in the 30 days after transplantation (left 99.1% vs. right 97.7%, log rank *p*-value = 0.005) (Figure 4A). Overall graft survival at 1 and 5 years was 97.4% (95% CI 96.9–97.9) and 89.6% (95% CI 88.5–90.6%) respectively. One-year overall graft survival was 97.7% (95% CI 97.1–98.2) for left kidneys and 96.1% (95% CI 94.3–97.3) for right kidneys. At 5 years, graft survival was 89.8% (95% CI 88.6–90.9) for left kidneys and 88.8% (95% CI 86.0–91.1) for right kidneys (Figure 4B).

Death-censored graft survival was lower for right kidney recipients in the first 30 days after transplantation (99.3% for left vs. 98.1% for right, log rank *p*-value = 0.005) (Figure 4C). The overall death-censored graft survival at 1 and 5 years was 98.4% (95% CI 98.0–98.8) and 93.7% (95% CI 92.8–94.4%) respectively. At 1 and 5 years, there was no significant difference between the death-censored graft survival of left and right kidney transplants (Figure 4D).

Patient survival was not significantly different between left and right kidney recipients at 30 days, 1 year or 5 years. Thirty days after transplantation, the survival of recipients of left and right kidneys was 99.7% (95% CI 99.5–99.9%) and 99.5% (95% CI 98.7–99.9%) respectively (Figure 4E). Overall patient survival at 1 and 5 years was 98.8% (95% CI 98.4–99.1) and 94.9% (95% CI 94.1–95.6%) respectively.

### Association Between Sides of the Kidney and Overall Graft Loss

With reference to the left kidney, the adjusted HR of overall graft loss within 30 days of transplantation was 2.02 [95% CI 1.06–3.86], *p* = 0.03 (Supplementary Figure S1A). Other risk factors for graft loss in the first 30 days included having more than one renal artery (aHR 2.05 [95%CI 1.07–3.91], *p* = 0.03) and the recipient having type 1 diabetes mellitus (aHR 4.26 [95% CI 1.51–12.04], *p* = 0.03) (Supplementary Figure S1A). After 30 days, the adjusted HR for overall graft loss among recipients who received right kidneys compared to the left was 0.97 [95% CI 0.80–1.19], *p* = 0.8 (Supplementary Figure S1B). In the frailty analysis, clustering for centres, the adjusted HR for left kidneys compared to right kidneys was 2.02 [95% CI 1.06–3.87] within 30 days and 0.97 [95% CI 0.80–1.19] after 30 days. The adjusted HRs for left kidneys compared to right kidneys, both within and after 30 days of transplantation, are unchanged after sensitivity analysis to account for the effect of transplant centres. Furthermore, both within and after 30 days of transplantation, the 5-year era in which the transplant occurred was not associated with the risk of graft loss.

### Association Between Sides of the Kidney and Death-Censored Graft Loss

Within the first 30 days after transplant, the adjusted HR for death-censored graft loss among recipients of right kidneys compared to left was aHR 2.14 [95% CI 1.05–4.34], *p* = 0.04) (Supplementary Figure S2A). Grafts with more than one renal artery were at increased risk of death-censored graft loss (aHR 2.11 [95% CI 1.04–4.28], *p* = 0.04) (Supplementary Figure S2A). After 30 days, right kidneys and more than one renal artery were no longer an independent risk factors for death-censored graft loss (Supplementary Figure S2B). In the frailty analysis, clustering for centres, the adjusted HR for left kidneys compared to right kidneys was 2.17 [95% CI 1.06–4.41] within 30 days and 0.89 [95% CI 0.67–1.16] after 30 days. Both within and after 30 days of transplantation, the 5-year era in which the transplant occurred was not associated with the risk of death-censored graft loss.

### Association Between Sides of the Kidney and All-Cause Death

Side was not an independent risk factor for patient survival either within or after the first 30 days (within the first 30 days, aHR 1.54 [95% CI 0.41–5.81], *p* = 0.5, and after the first 30 days aHR 1.22 [95% CI 0.95–1.57], *p* = 0.1) (Supplementary Figures S3A,S3B). In the frailty analysis, clustering for centres, the adjusted HR for left kidneys compared to right kidneys was 1.54 [95% CI 0.41–5.81] within 30 days and 1.22 [95% CI 0.94–1.57] after 30 days. Both within and after 30 days of transplantation, the 5-year era in which the transplant occurred was not associated with the risk of patient survival.

## Discussion

This large registry-based study demonstrates that adult recipients of primary right living donor kidneys have a two-fold increased risk of DGF, and graft loss and death-censored graft loss within the first 30 days of transplantation. Primary non-function accounted for 9% of left kidneys and 36% of right kidneys that were lost in the first 30 days after transplantation. Patient survival and graft survival beyond 30 days were not associated with living donor kidney side.

The association between living donor kidney side and recipient outcomes has mostly been studied previously in small, single-centre studies with disparate results, complicating the debate as to whether more right-sided nephrectomies should be undertaken. An OPTN registry-based retrospective analysis from 2000 to 2009 reached similar conclusions to our study, with lower effect sizes; right living donor kidneys experienced a 1.4 (95% CI 1.2–1.5) increased risk of DGF and a 1.1 (95% CI 0.85–1.5) increased risk of graft loss (2). An earlier ANZDATA analysis investigating DGF identified right sided kidneys as a risk factor (14). In contrast, two meta-analyses have shown that right laparoscopic living donor kidneys were not associated with increased rates of DGF, after sensitivity analysis, or graft loss at 1 year (10, 13).

Our multi-centre and registry-based study had sufficient power to examine differences in DGF, patient survival and graft survival. Furthermore, the findings of our study corroborated prior work that compared outcomes between transplanted left and right deceased donor kidneys (9). The inferior results of transplanted right deceased donor kidneys in that study may be attributed to technical challenges, with a recommendation that transplanting teams optimise allocation of surgical expertise (9). However, in this study the inferior outcomes of transplanting right-sided living donor kidneys could not be proven to directly relate to surgical challenges associated with its transplantation. There are some important differences to note, for example, unlike deceased donor procedures, the transplantation of living donor kidneys are typically undertaken as elective day-time procedures in optimally prepared recipients and carefully selected donors. Furthermore, the low incidence of graft loss in the first 30 days after transplant likely limited our analysis of cause-specific graft loss. Equally, this low rate of graft loss by international registry standards, reflects the good outcomes of kidney transplantation in Australia and New Zealand (20, 21).

However, the demonstrated higher incidence of DGF and PNF-related graft loss in right living donor kidneys in the first 30 days after transplantion supports increased surgical challenges associated with transplanting right living donor kidneys. In the first 30 days, 64.3% of right kidneys that were lost were lost due to either primary non-function or technical causes compared to 59.4% of left kidneys that were lost. Expanding data collection to include important factors such as vascular anastomosis times, and intra-operative and post-operative complications could help determine if there are increased surgical challenges with right living donor kidney transplantation. For example, although anastomosis times were not captured by the ANZDATA registry, right deceased donor kidneys have been shown to have longer anastomosis times (22).

Our study has a number of limitations. Indication bias remains a possibility. We were unable to account for inter-centre decision-making variations that might influence outcomes such as indications for right donor nephrectomies in preference to left. This clinical decision evaluates the risk of surgery on either side and aims to maximise the residual renal function of the donor. However, the shared frailty models demonstrated minimal changes to the estimates when accounting for centre-specific random effects. Even though there were multiple confounding factors adjusted for in the analyses, there are likely to be several unmeasured and residual confounders, such as differential kidney function of the living donor kidneys and individual surgeons’ volumes and expertise. The definition of DGF changed from the need for dialysis within 72 h after transplantation to the need for dialysis within 7 days in 2017. We defined DGF as the need for dialysis within 7 days, therefore this may lead to an underestimation of the overall incidence of DGF. Adjusting for the era of the transplantation in the model did not change the risk factor profile. However, only 2 years of data were captured using the revised ANZDATA Registry definition. Additionally, the outcome ascertainment bias is unlikely to be differential between recipients of left and right living donor kidneys, as the proportion of right kidneys transplanted each year was relatively stable. Strengths of this study are the large cohort with few missing values and cases lost to follow up and the minimal risk of selection bias as the study population represents all primary adult recipients of kidney transplants in Australia and New Zealand.

The time period of this ANZDATA based study corresponded to the progressive uptake of laparoscopic donor nephrectomy. The driving force behind this was a consumer-driven preference by prospective living kidney donors and their referring nephrologists to avoid open surgery where possible. In the 15 year study period, 10 of 27 centres ceased to provide a living donor kidney transplantation service, likely driven by their inability to provide laparoscopic surgical expertise. The higher percentage of right nephrectomies performed by open or hand assisted laparoscopic surgery and low overall rates of right nephrectomies likely reflected the increasing uptake of laparoscopic nephrectomies and hesitancy to undertake right laparoscopic nephrectomies during this learning phase, particularly in the early part of the study. Importantly, this careful approach resulted in equivalent recipient outcomes for laparoscopic and open donor surgery, as the type of operation was not a risk factor for graft or patient survival. Furthermore, the era of transplantation was not a risk factor for inferior recipient outcomes. Centres with low volumes of transplants were not excluded in the study, as they remained important data points, and despite the variation in transplant centre volume, the shared frailty models demonstrated minimal changes to the estimates when accounting for centre-specific random effects. Equally, the use of right living donor kidneys (17.2%), which is high by international standards, suggests that it would have been uncommon for recipients in Australia and New Zealand to be denied the opportunity to be transplanted with a living donor kidney because their transplant centre had been reluctant to tackle either donation or transplantation of a right-sided donor kidney.

Robot-assisted surgery may have an emerging role in living kidney transplantation and the impact of the side of the living donor kidney should be studied in this context. There has been increasing uptake of robot-assisted kidney transplantation with initial studies indicating that it is non-inferior to open kidney transplantation (23) and feasible with multiple vessel grafts (24). The shorter renal vein of right kidneys is particularly an issue in obese recipients and recipients with narrow pelvises in the setting of the traditional open approach. The magnification and dexterity possible with the robotic platform is particularly advantageous in these situations as it facilitates the formation of tension-free vascular anastomosis even in the case of short renal veins. However, implementation of this technique requires appropriate training and a team with extensive experience in both robotic surgery and open transplantation.

In summary, our results indicate that recipients of right living donor kidneys may have an increased risk of DGF and graft loss in the first 30 days after transplantation. The implication is that the technical challenges of transplanting a right living donor kidney are real, but not to the extent that right-sided kidneys should be excluded, particularly in light of the limitations addressed above. The prospective donor of a right kidney and the recipient should be informed but also reassured that the differences between left and right living donor kidneys are relatively small, confined to the early post-operative period and are similar to those seen in recipients of left or right deceased donor kidneys (9). Nevertheless, the increased risks associated with receiving a right kidney should be factored into trial-based analyses and published living donor kidney transplant outcomes of individual transplant centres. The underlying mechanisms of the observed findings of this study may be clarified by prospective studies or analyses of data at large transplant centres, with the availability of additional variables such as anastomosis times, pre-operative differential kidney function, intra-operative complications and other provider-related factors. Overall, a patient in need of kidney transplantation should not be denied this opportunity only because of reluctance to use a right-sided living donor kidney.

## Data Availability

The data analyzed in this study is subject to the following licenses/restrictions: Raw data were generated at the Australia and New Zealand Dialysis and Transplant Registry (ANZDATA). Data can be accessed through application to ANZDATA. Requests to access these datasets should be directed to requests@anzdata.org.au.

## Abbreviations

aHR, adjusted hazard ratio; ANZDATA, Australia and New Zealand Dialysis and Transplant Registry; CMV, cytomegalovirus; DGF, delayed graft function; EBV, Epstein-Barr virus; GN, glomerulonephritis; HLA, human leukocyte antigen; PCKD, polycystic kidney disease; PNF, primary non-function.
