# Supplementary material for: Association Between Side of Living Kidney Donation and Post-Transplant Outcomes
Source: Transpl Int. 2022 Apr 4;35:10117. doi: 10.3389/ti.2022.10117 (PMC9013757; doi:10.3389/ti.2022.10117)
Supplement: Supplementary file 1 [file DataSheet1.docx]

**Supplemental Figures**

**
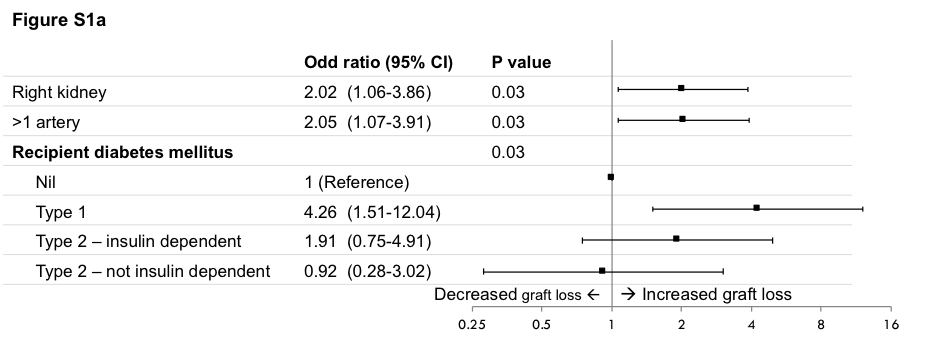
**

**Figure S1a:** Risk factors for graft loss, within 30 days of transplant.


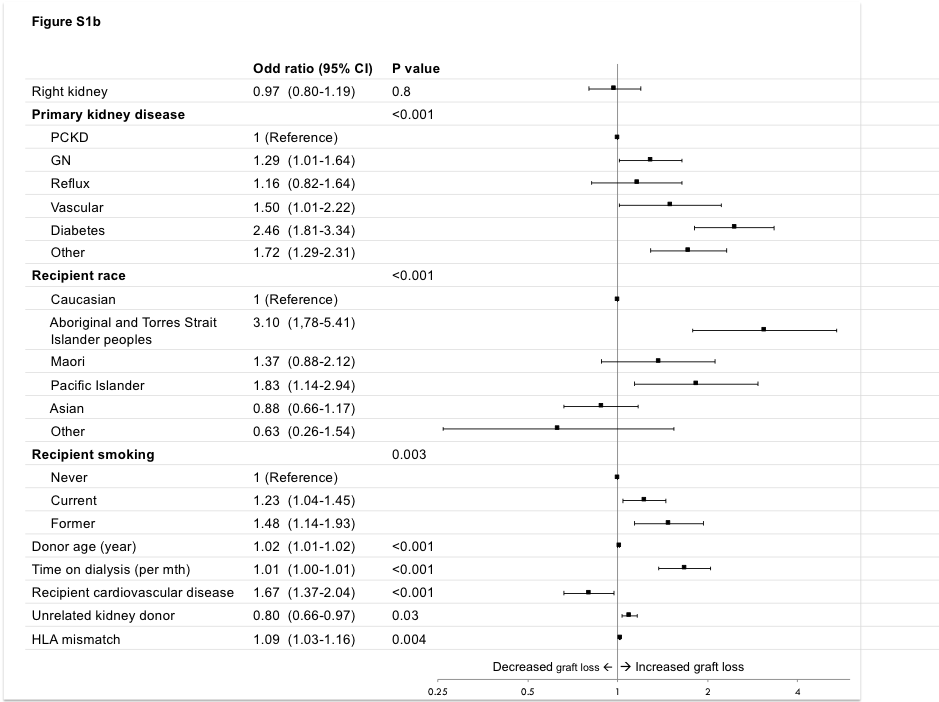


**Figure S1b:** Risk factors for graft loss, after first 30 days. GN, glomerulonephritis; PCKD, polycystic kidney disease.


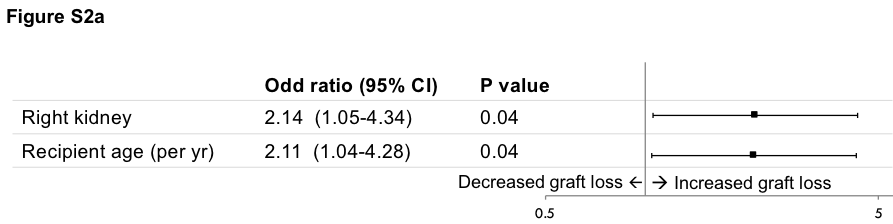


**Figure S2a:** Risk factors for death-censored graft loss, within 30 days of transplant.


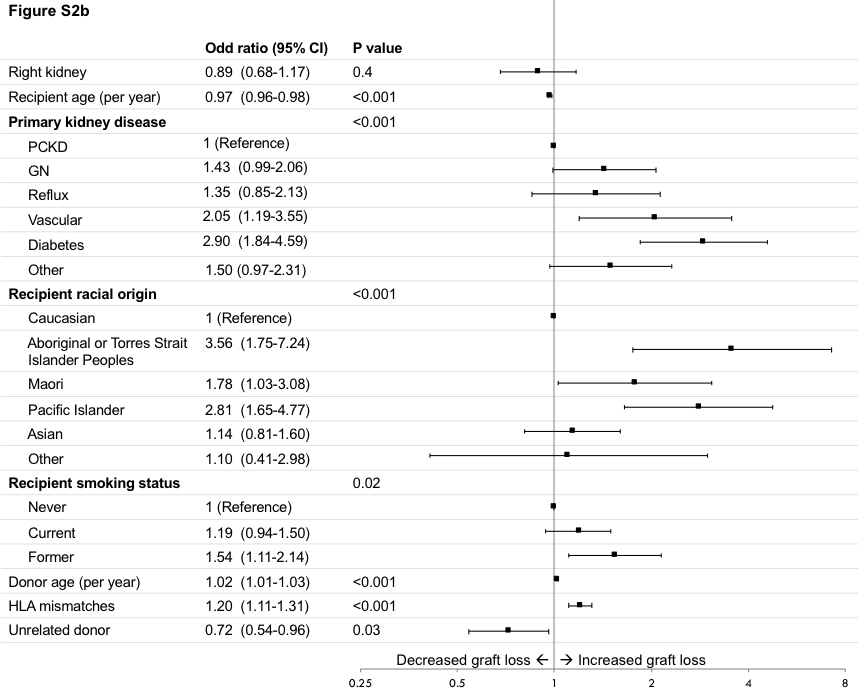


**Figure S2b:** Risk factors for death-censored graft loss, after 30 days. GN, glomerulonephritis; PCKD, polycystic kidney disease.


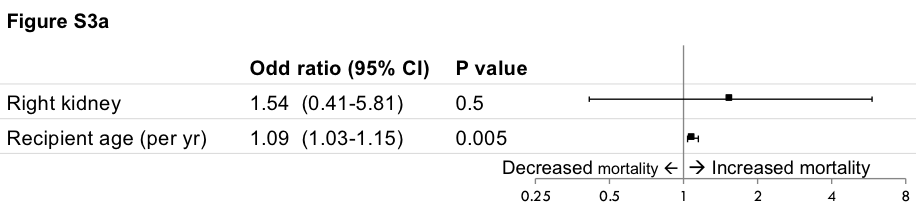


**Figure S3a:** Risk factors for all-cause mortality, within 30 days of transplant.


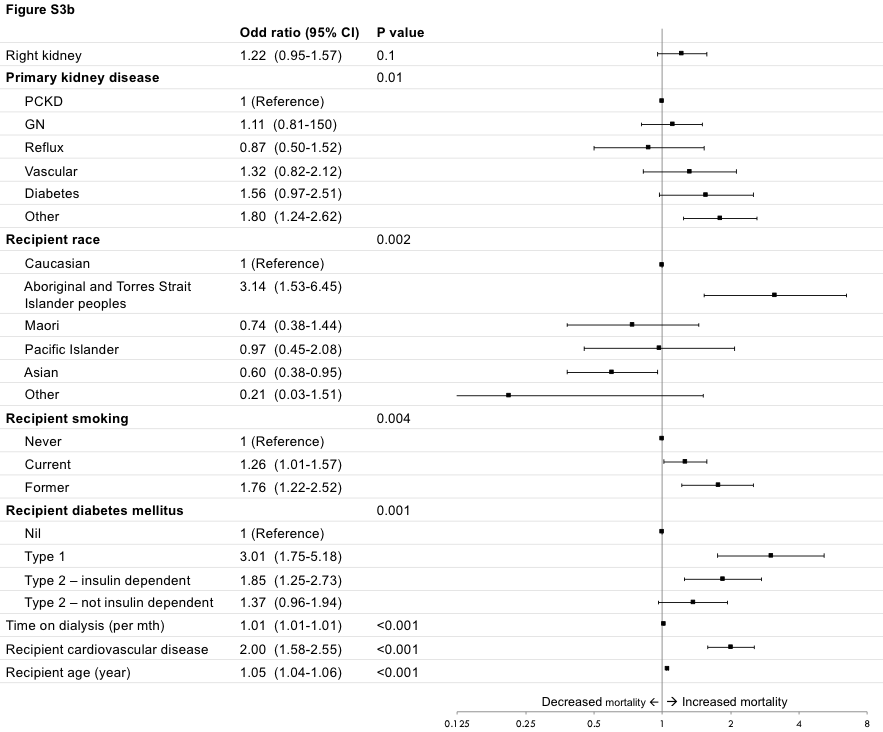


**Figure S3b:** Risk factors for all-cause mortality, after first 30 days. GN, glomerulonephritis; PCKD, polycystic kidney disease.
